# Global burden of lung cancer in adolescents and adults aged 15–45: analysis of the global burden of disease study (1990–2021)

**DOI:** 10.3389/fmed.2025.1600662

**Published:** 2025-06-25

**Authors:** Ziqi Han, Zhigang Zhu, Zhiqiang Zhang, Jiayang Dong, Xinyue Yang, Jing Feng

**Affiliations:** ^1^Department of Respiratory, Tianjin Medical University General Hospital, Tianjin, China; ^2^Department of Cardiology, Tianjin Medical University General Hospital, Tianjin, China

**Keywords:** global burden of disease, lung cancer, adolescents and young adults, epidemiology, risk factors

## Abstract

**Background:**

Lung cancer is a leading cause of cancer-related deaths around the world, but its impact on young and middle-aged individuals (aged 15–45 years) is less understood. The aim of this study was to assess the global burden of lung cancer in those aged 15–45 years during 1990–2021.

**Methods:**

This study estimated global trends in prevalence, mortality, and disability-adjusted life years (DALYs) associated with lung cancer in this age group. The Global Burden of Disease Study 2021 was utilized to analyze data from 1990 to 2021. We examined variations by sex, age subgroup, sociodemographic index (SDI), and region and assessed key risk factors contributing to DALYs.

**Results:**

Our findings reveal that while the number of lung cancer cases aged 15–45 increased by 22.1%, the age-standardized prevalence decreased by 23.9%; age-standardized mortality and DALYs declined significantly over the past three decades. The decrease was more evident among men than women, despite men having a higher overall disease burden. In high-SDI countries, prevalence and DALYs experienced substantial decreases, whereas low-SDI countries experienced increases, particularly among women. East Asia demonstrated a high disease burden. Smoking was identified as the most prominent risk factor, especially in high-middle SDI countries. Household air pollution and ambient particulate matter pollution came after it.

**Conclusion:**

The study underscores the effectiveness of tobacco control measures and early screening in reducing lung cancer burden among young and middle-aged individuals. Nevertheless, the upward trends in low - SDI countries emphasize the necessity for interventions that are specifically aimed at environmental risk factors and smoking cessation initiatives. These findings provide valuable insights for policymakers and healthcare providers aiming to implement strategies to further cut down the global burden of lung cancer in the younger population.

## 1 Introduction

Globally, lung cancer still ranks as the leading cause of death due to cancer (1). Based on the data from GLOBOCAN 2020, it was estimated that 2.2 million new cases of lung cancer emerged, and 1.8 million lung cancer-related deaths occurred (2). Lung cancer is relatively rare before the age of 50, with fewer than 3.5% of patients are under 45 years old in China (3). As is known, smoking - related lung cancers still make up the largest part of all lung cancer diagnoses, and risk increases with age thereafter. Women are less affected compared to men (4). With increased awareness of lung cancer and active tobacco control, attention is shifting toward rarer forms of lung cancer that were previously overlooked. This includes lung cancer in young and middle-aged individuals, which imposes a significant burden on society and the patients’ families. Several studies have explored lung cancer in young patients, indicating that the proportion of women and non - smokers is on the rise (5, 6). Furthermore, these patients typically experience symptoms for a more extended period. They have a greater likelihood of having adenocarcinoma instead of squamous cell carcinoma, and are more often diagnosed at an advanced stage (7). There is still debate over whether the outcomes of younger patients are similar to, better than, or worse than those of older patients. Moreover, recent research has indicated that young non - small cell lung cancer (NSCLC) patients possess a higher number of driver mutations compared to older patients (8). In recent years, an emerging disease called lung cancer in individuals who have never smoked (LCINS) has challenged people’s understanding of traditional lung cancer cases (9). This has led us to transform our attention on lung cancer caused by genetic or environmental factors other than tobacco smoke exposure.

The 15–45 age group deserves specific epidemiological focus for several compelling reasons: (1) Economic impact - lung cancer during peak productive years results in disproportionate societal burden; (2) Biological distinctiveness - younger patients exhibit different molecular profiles with higher rates of driver mutations; (3) Emerging risk patterns - increasing lung cancer in never-smokers, particularly women, suggests distinct etiological pathways; and (4) Healthcare gaps - younger patients often face delayed diagnosis due to low clinical suspicion and lack of age-appropriate guidelines. Against this background, we investigated the comprehensive burden of lung cancer among individuals aged 15–45 years from 1990 to 2021. Our analysis considered global, regional, and national levels, along with variations by social development, age, and sex. We also evaluated the elements potentially affecting the DALYs in young and middle-aged people with lung cancer.

## 2 Materials and methods

### 2.1 Data collection

For our research based on the Global Burden of Disease Study 2021, we utilized cross-sectional information regarding 369 diseases and 87 risk factors, among which was lung cancer. This data covered 204 countries and regions spanning from 1990 to 2021. We focused on lung cancer in individuals aged 15–45, examining prevalence, mortality, and DALYs by location, age, and sex, with 95% uncertainty intervals. Data covered six age groups (15–19, 20–24, 25–29, 30–34, 35–39, and 40–45 years) for both sexes across 21 regions. The DALYs attributed to risk factors and the sociodemographic index (SDI) were computed. The SDI, a comprehensive metric combining education, income, and fertility, was classified into five tiers: low, low-middle, middle, high-middle, and high. The analysis conducted herein underscores the effects of certain risk factors and the socioeconomic conditions on the global burden of lung cancer. Ethical approval and informed consent were not required for this study because the data is publicly available.

### 2.2 Statistical analysis

A descriptive analysis was performed to characterize the burden of lung cancer among adults aged 15–45 years on a global scale. We compared the age-standardized prevalence rate (per 100,000 population), age-standardized mortality rate (per 100,000 population), and age-standardized DALYs rate (per 100,000 population) of lung cancer across different age groups, sexes, regions, and countries. Using data on lung cancer and associated risk factors from the Global Burden of Disease Study, we calculated age-standardized rates and corresponding 95% confidence intervals (CIs) based on the world standard population reported in the 2017 study. Additionally, we estimated average annual percentage changes (AAPCs) through joinpoint regression to measure temporal trends.

All statistical analyses were conducted using GraphPad Prism (version 8.0), the Joinpoint Regression program (version 5.0.2), and R (version 4.2.3).

## 3 Results

### 3.1 Global trends

On a global scale, the count of lung cancer cases among individuals aged 15–45 increased by 22.1%, from 103,025 in 1990 to 125,815 in 2021. However, the age-standardized prevalence rate in this group declined by 23.9%, from 4.6 to 3.5 per 100,000, showing an annual tendency of −0.91% (Table 1). Similarly, the percentage of lung cancer cases in this group relative to total cases dropped from 7.4 to 3.9% (Supplementary Figure 1A). The age-standardized prevalence of lung cancer in those aged 15–45 was significantly lower than in all age groups, indicating it primarily affects those over 45 (Supplementary Figure 2). The age-standardized mortality rate of lung cancer of this age group decreased by 40%, from 2.2 to 1.3 per 100,000, with an annual decline of −1.63% (Supplementary Table 1). The disease burden, measured by DALYs, also fell by 39%, from 113.7 to 69.3 per 100,000, showing an annual alternation of −1.6% (Supplementary Table 1). These trends highlight a decreasing burden of lung cancer among younger populations.

**Table 1:** Age standardized prevalence and AAPC of Lung Cancer in people aged 15–45 years at global and regional level, 1990–2021.

Location	Prevalence (95% UI)				AAPC (95% CI)
	No of people in 1990	ASR in 1990 (per 100,000)	No of people in 2021	ASR in 2021 (per 100,000)	
Global	103025.5	4.6	125,815	3.5	−0.91
	(96129.3 to 110410.8)	(4.3 to 5)	(112551.6 to 139530.4)	(3.1 to 3.9)	(−1.09 to −0.7)
Sex					
Female	35070.8	3.2	52763.9	3	−0.25
	(31369.4 to 39,414)	(2.9 to 3.6)	(46130.4 to 60865.9)	(2.6 to 3.4)	(−0.35 to −0.2)
Male	67954.8	6	73051.1	4	−1.3
	(62349.5 to 74568.5)	(5.5 to 6.6)	(63,085 to 83950.3)	(3.5 to 4.6)	(−1.54 to −1.1)
Age group (years)					
15–19	2057.3	0.4	1630.3	0.3	−1.35
	(1870.1 to 2,307)	(0.4 to 0.4)	(1497.3 to 1775.9)	(0.2 to 0.3)	(−1.44 to −1.3)
20–24	3039.1	0.6	2946.1	0.5	−0.72
	(2753.2 to 3349.2)	(0.6 to 0.7)	(2704.7 to 3201.7)	(0.5 to 0.5)	(−0.9 to −0.5)
25–29	7,484	1.7	8,200	1.4	−0.65
	(6884.7 to 8172.2)	(1.6 to 1.8)	(7439.1 to 8987.1)	(1.3 to 1.5)	(−0.95 to −0.3)
30–34	16461.4	4.3	22234.8	3.7	−0.42
	(15306.6 to 17699.3)	(4 to 4.6)	(19842.2 to 24739.3)	(3.3 to 4.1)	(−0.91 to 0.1)
35–39	31323.9	8.9	36738.8	6.6	−1.01
	(29186.5 to 33620.8)	(8.3 to 9.5)	(32789.3 to 40933.4)	(5.8 to 7.3)	(−1.21 to −0.8)
40–45	42659.9	14.9	54064.9	10.8	−1.04
	(40128.3 to 45262.3)	(14 to 15.8)	(48278.9 to 59,893)	(9.7 to 12)	(−1.23 to −0.9)
SDI level					
High	28570.2	6.5	22679.3	4.5	−1.2
	(27690.9 to 29534.8)	(6.3 to 6.7)	(21,361 to 24117.2)	(4.2 to 4.8)	(−1.5 to −0.9)
High-middle	35362.4	7.3	39343.9	6.2	−0.49
	(32008.7 to 39010.3)	(6.6 to 8)	(33335.4 to 45955.2)	(5.3 to 7.3)	(−0.81 to −0.2)
Middle	30,958	4.3	45,223	3.9	−0.36
	(27523.7 to 34960.4)	(3.8 to 4.9)	(38108.4 to 52176.5)	(3.3 to 4.5)	(−0.57 to −0.2)
Low-middle	6591.9	1.5	14,664	1.7	0.34
	(5835.8 to 7572.7)	(1.4 to 1.8)	(13028.8 to 16726.2)	(1.5 to 1.9)	(0.19 to 0.5)
Low	1,431	0.9	3814.8	0.9	0.2
	(1,181 to 1822.3)	(0.7 to 1.1)	(3110.2 to 4687.9)	(0.8 to 1.1)	(0.03 to 0.4)

AAPC, average annual percentage change; CI, confidence interval; SDI, sociodemographic index; No, number; UI, uncertainty interval; ASR, Age-standardized rate.

### 3.2 Global trends by sex

In the period from 1990 to 2021, lung cancer cases among individuals aged 15–45 increased globally for both sexes, rising by 50.5% for women (from 35,071 to 52,764) and 7.5% for men (from 68,000 to 73,000). The increase was greater in women, but men still had more cases (Table 1). Age-standardized prevalence decreased for both genders, with a sharper decline in men (from 6 to 4 per 100,000, AAPC −1.3%) compared to women (from 3.2 to 3 per 100,000, AAPC −0.25%). During the same period, age-standardized mortality also fell more in men (AAPC −1.85%) than in women (−1.17%) (Supplementary Table 1).

**Figure 1:**
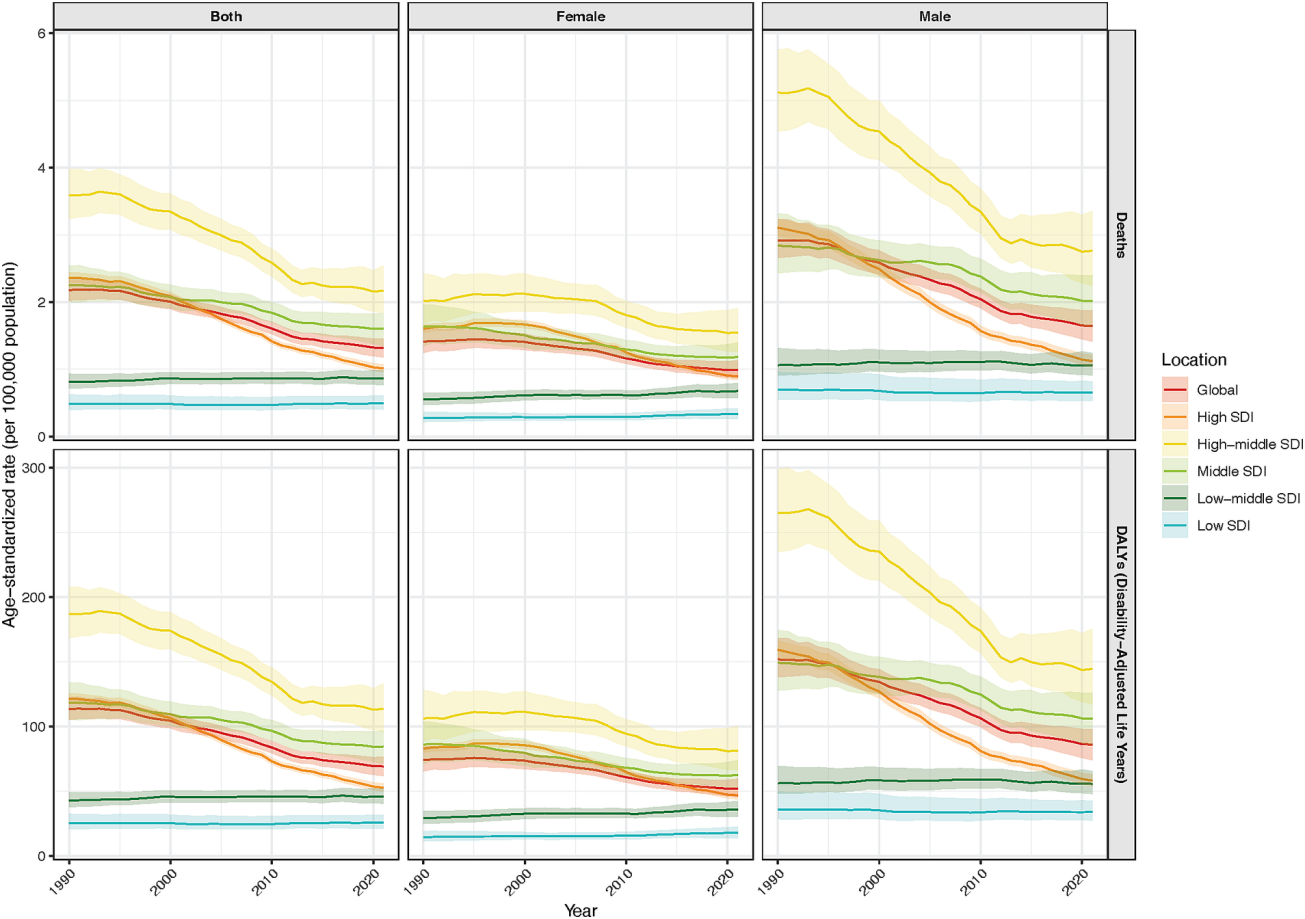
Analysis of the temporal trends in age-standardized mortality and disability-adjusted life years of lung cancer among young and middle - aged populations from 1990 to 2021, across global and socio - demographic index levels, differentiated by sex.

In the period from 1990 to 2021, age standardized DALY declined faster for men, with an annual decrease of −1.83%, though men consistently had higher DALYs than women (152 vs. 74 in 1990; 86 vs. 52 in 2021) (Supplementary Table 1; Figure 1). Men aged 15–45 had a higher lung cancer burden, but their reduction over 32 years was greater than women’s. Among women in low and low-middle SDI regions, mortality and DALYs increased significantly (Supplementary Figure 3).

### 3.3 Global trends by age subgroup

From 1990 to 2021, lung cancer cases declined in the 15–19 and 20–24 age groups but increased in older subgroups (Table 1; Supplementary Figure 4). Age-standardized prevalence and mortality decreased across all subgroups, with the sharpest declines in the 15–19 group (AAPC -1.35% for prevalence, −1.83% for mortality) (Supplementary Table 1). In 2021, DALYs increased with age, from 8.6 per 100,000 in the 15–19 group to 236.4 per 100,000 in the 40–45 group.

### 3.4 Global trends by sociodemographic index

**Figure 2:**
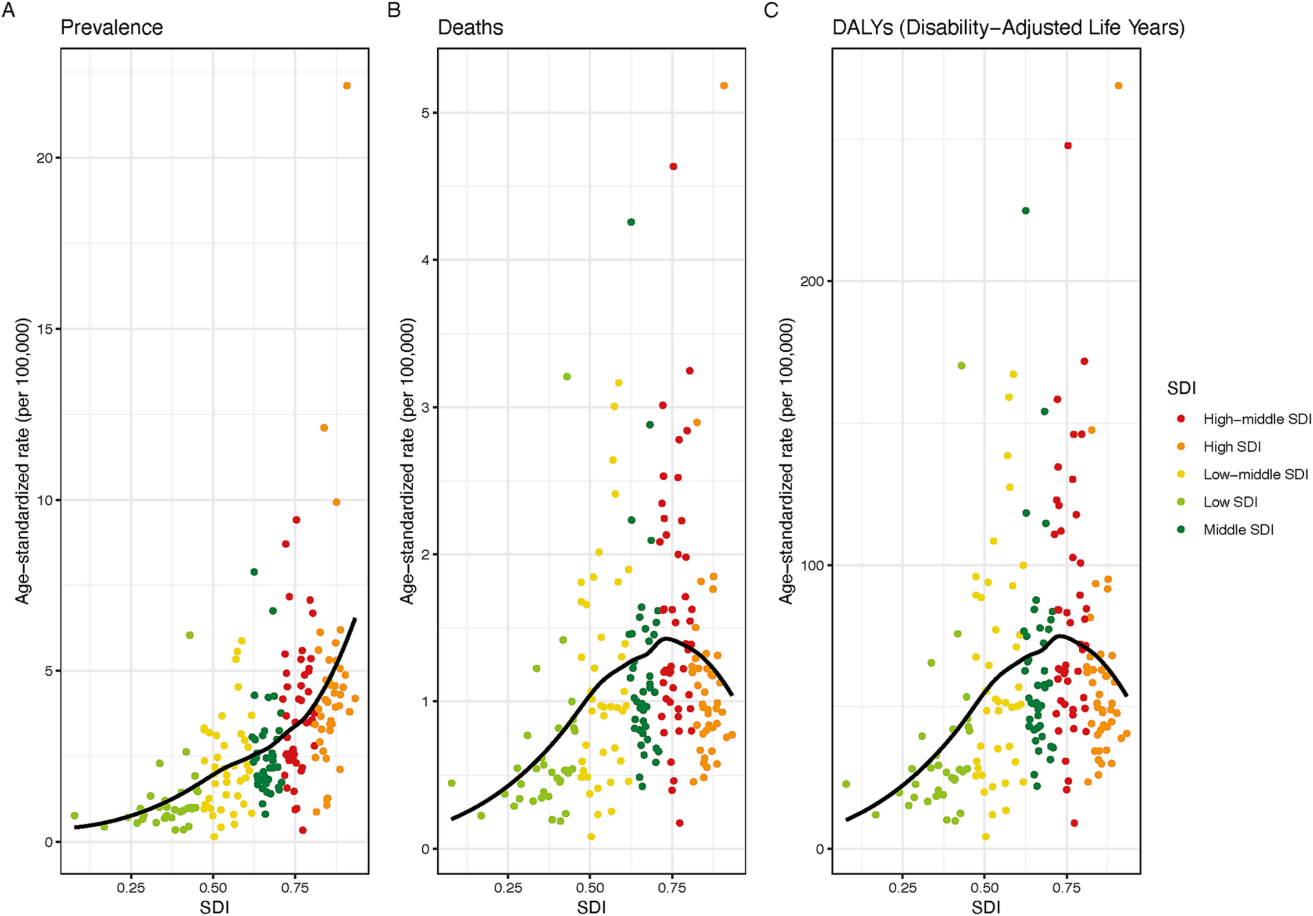
The rates of prevalence **(A)**, mortality **(B)**, and disability - adjusted life years **(C)** of lung cancer among young and middle - aged individuals in 204 countries in 2021, based on the socio - demographic index.

From 1990 to 2021, lung cancer trends among individuals aged 15–45 varied by sociodemographic index (SDI). High SDI countries saw a 20.6% decline in cases, while low-SDI regions experienced the sharpest growth (Table 1; Supplementary Figure 5). Age-standardized prevalence declined in high, high-middle, and middle SDI countries (AAPC −1.2, −0.49%, −0.36%, respectively) but rose in low-middle and low SDI countries (AAPC 0.34%). High SDI countries experienced the greatest decline in mortality (AAPC −2.73%) and DALYs (AAPC −2.68%). A distinct pattern emerged where DALYs decreased markedly when SDI exceeded 0.7 (Figure 2).

### 3.5 Regional trends

From 1990 to 2021, lung cancer prevalence among individuals aged 15–45 increased in five regions: East Asia, Oceania, South Asia, Southeast Asia, and Western Sub-Saharan Africa. East Asia (AAPC 0.62%), South Asia (AAPC 0.59%), and Western Sub-Saharan Africa (AAPC 0.4%) showed the most rapid increases, while 16 regions experienced declines (Table 2; Supplementary Figure 6). The largest decreases were in Central Asia (AAPC −3.14%), high-income North America (AAPC −2.54%), and Southern Latin America (AAPC −2.35%) (Supplementary Figure 6). In 2021, the age-standardized prevalence was highest in East Asia (9 per 100,000) (Table 2). Moreover, following sex stratification in 2021, East Asia continued to exhibit the highest age-standardized prevalence (Supplementary Table 2).

**Table 2:** Age-standardized prevalence of lung cancer in young and middle-aged people and their AAPCs from 1990 to 2021 at regional levels.

Regions	Prevalence (95% UI)				AAPC (95% CI)
	No of people in 1990	ASR in 1990 (per 100,000)	No of people in 2021	ASR in 2021 (per 100,000)	
Andean Latin America	398.5 (325.9 to 490.7)	2.7 (2.2 to 3.3)	666.1 (495.8 to 864.5)	2.2 (1.6 to 2.8)	−0.72 (−1.64 to 0.2)
Australasia	483.2 (417.2 to 561.8)	4.7 (4.1 to 5.5)	650.2 (527.4 to 796.6)	4.6 (3.8 to 5.7)	−0.14 (−0.99 to 0.7)
Caribbean	536.2 (476 to 599.1)	3.8 (3.3 to 4.2)	582.2 (484.6 to 692.7)	2.8 (2.3 to 3.3)	−1.03 (−1.58 to −0.5)
Central Asia	1613.1 (1498.7 to 1724.4)	6.3 (5.9 to 6.8)	1080.5 (950.2 to 1,232)	2.4 (2.1 to 2.7)	−3.14 (−3.58 to −2.7)
Central Europe	4337.5 (4091.9 to 4605.3)	7.2 (6.7 to 7.6)	2092.7 (1878.5 to 2303.2)	3.7 (3.3 to 4.1)	−2.08 (−2.38 to −1.8)
Central Latin America	1,560 (1480.5 to 1642.8)	2.4 (2.3 to 2.6)	2039.1 (1771.4 to 2,346)	1.7 (1.5 to 2)	−1.11 (−1.56 to −0.7)
Central Sub-Saharan Africa	203.5 (134.9 to 301.7)	1.2 (0.8 to 1.8)	559.1 (368.1 to 869.2)	1.1 (0.8 to 1.8)	−0.13 (−0.26 to 0)
East Asia	40518.3 (34433.3 to 47047.3)	7.2 (6.1 to 8.4)	58323.7 (46728.1 to 71161.1)	8.6 (6.9 to 10.5)	0.62 (0.39 to 0.9)
Eastern Europe	7476.1 (7062.7 to 7,975)	7.1 (6.7 to 7.6)	4020.1 (3608.5 to 4488.8)	3.8 (3.4 to 4.2)	−2.03 (−2.39 to −1.7)
Eastern Sub-Saharan Africa	544.2 (444.5 to 695.9)	1 (0.8 to 1.2)	1370.7 (1104.9 to 1745.5)	0.9 (0.7 to 1.1)	−0.24 (−0.33 to −0.1)
High-income Asia Pacific	4672.6 (4314.7 to 5087.7)	5.3 (4.9 to 5.8)	3737.5 (3216.2 to 4320.2)	4.7 (4 to 5.4)	−0.57 (−0.77 to −0.4)
High-income North America	11229.5 (10869.9 to 11596.9)	7.8 (7.5 to 8)	5626.3 (5356.5 to 5922.8)	3.5 (3.3 to 3.7)	−2.54 (−2.7 to −2.4)
North Africa and Middle East	7355.3 (5726.3 to 9365.3)	3 (2.3 to 3.8)	13098.8 (11107.7 to 15347.7)	2.1 (1.8 to 2.5)	−1.15 (−1.31 to −1)
Oceania	64.7 (42.4 to 102.2)	2.6 (1.7 to 4.1)	170.3 (112.9 to 258.9)	2.9 (1.9 to 4.3)	0.32 (0.08 to 0.6)
South Asia	9,660 (8329.5 to 11460.5)	1.1 (1 to 1.4)	23955.6 (20516.4 to 27872.2)	1.4 (1.2 to 1.6)	0.59 (0.37 to 0.8)
South-East Asia Region	5632.9 (4,822 to 6419.6)	3 (2.6 to 3.5)	11432.4 (9131.1 to 13880.6)	3.4 (2.7 to 4.1)	0.3 (0.15 to 0.4)
Southern Latin America	1109.9 (963.3 to 1283.2)	5.3 (4.6 to 6.1)	826.2 (703.6 to 967)	2.6 (2.2 to 3)	−2.35 (−2.8 to −1.9)
Southern Sub-Saharan Africa	746.7 (621.6 to 878.8)	4 (3.3 to 4.7)	964.6 (814.7 to 1141.4)	2.55 (2.1 to 3)	−1.45 (−2.12 to −0.8)
Tropical Latin America	1611.6 (1516.5 to 1719.1)	2.5 (2.4 to 2.7)	2,328 (2183.2 to 2489.8)	2 (1.9 to 2.2)	−0.69 (−1 to −0.4)
Western Europe	11524.4 (10810.2 to 12232.5)	6.4 (6 to 6.8)	10035.5 (9100.9 to 11073.4)	5.3 (4.8 to 5.8)	−0.66 (−0.94 to −0.4)
Western Sub-Saharan Africa	254.8 (206.5 to 313.7)	0.4 (0.3 to 0.5)	782.3 (576.2 to 1034.3)	0.5 (0.3 to 0.6)	0.4 (0.35 to 0.5)

AAPC, average annual percentage change; CI, confidence interval; SDI, sociodemographic index; No, number; UI, uncertainty interval; ASR, Age-standardized rate.

Most regions saw reductions in DALYs during 1990–2021, except Oceania, South Asia, South-East Asia, and Western Sub-Saharan Africa. High-income North America (AAPC −3.48%), Central Asia (AAPC −3.38%), and Southern Latin America (AAPC −2.94%) exhibited the steepest drops (Supplementary Figure 6). In 2021, East Asia had the highest DALYs (157 per 100,000), followed by South-East Asia (88 per 100,000) and Oceania (81 per 100,000) (Supplementary Table 3). In 2021, East Asia had the highest age-standardized DALYs and mortality after sex stratification (Supplementary Table 2). Despite this, the reduction in mortality and DALYs suggests effective lung cancer control in East Asia. However, significant increases in DALYs among women were observed in South Asia, Oceania, Western Sub-Saharan Africa, Southeast Asia, Eastern Sub-Saharan Africa, and Central Latin America, highlighting the need for early screening for women aged 15–45 in these regions (Supplementary Figure 7).

### 3.6 National trends

**Figure 3:**
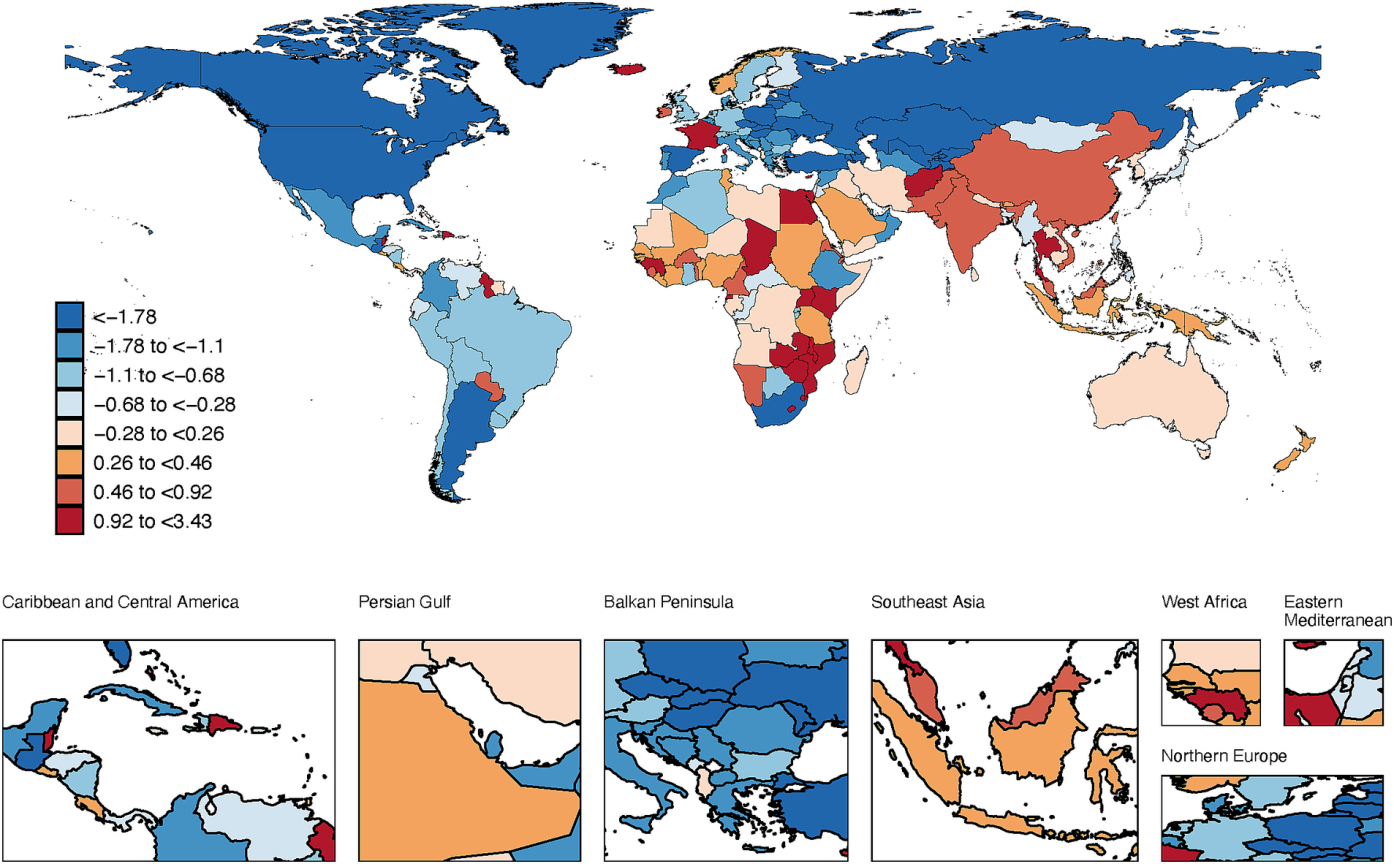
The map illustrates the average annual percentage change in global prevalence of lung cancer among people aged 15–45 years, 1990–2021.

At the national level, Kazakhstan showed the largest decrease in age-standardized prevalence (AAPC −3.95%), while Lesotho had the greatest increase (AAPC 3.43%). In 2021, Iceland had the highest prevalence (10 per 100,000) and Monaco the highest DALYs (269 per 100,000) (Figure 3, Supplementary Figures 8, 9; Supplementary Table 4).

### 3.7 Risk factors

From 1990 to 2021, five main risk factors were identified for lung cancer DALYs in people aged 15–45: smoking, household air pollution from solid fuels, ambient particulate matter pollution, a diet low in fruits, and secondhand smoke exposure. Smoking was the predominant contributor, accounting for 23 DALYs per 100,000 in 2021. The impact of smoking varied across sociodemographic index subgroups, with the most marked burden in high-middle SDI countries, where it contributed to 46 DALYs per 100,000 (Table 3).

**Table 3:** Main risk factors for age standardized lung cancer related DALYs, among people aged 15–45, 1990–2021.

Risk factors by SDI	Age standardized DALYs (per 100,000)(95% UI)	
	1990	2021
Smoking		
Global	51.96 (47.56 to 57.43)	23.25 (19.98 to 27.19)
High SDI	70.56 (65.99 to 74.81)	21.24 (19.15 to 23.29)
High-middle SDI	91.33 (81.6 to 102.67)	46.16 (38.25 to 55.62)
Middle SDI	45 (38.47 to 53.38)	26.73 (21.34 to 32.84)
Low-middle SDI	13.4 (11.35 to 16.46)	10.44 (8.73 to 12.43)
Low SDI	5.12 (3.94 to 7.06)	4.14 (3.17 to 5.48)
Household air pollution from solid fuels		
Global	19.32 (12.27 to 27.31)	4.57 (2.06 to 9.28)
High SDI	1.9 (0.63 to 4.44)	0.03 (0 to 0.22)
High-middle SDI	28.9 (17.4 to 42.86)	1.53 (0.08 to 7.92)
Middle SDI	30.57 (19.62 to 42.68)	4.59 (1.08 to 12.27)
Low-middle SDI	12.18 (8.03 to 16.59)	8.04 (4.5 to 12.69)
Low SDI	8.78 (5.74 to 12.37)	7.82 (4.9 to 11.19)
Ambient particulate matter pollution		
Global	11.79 (6.97 to 17.72)	10.67 (6.51 to 15.2)
High SDI	14.93 (8.52 to 22.89)	5.56 (3.37 to 7.85)
High-middle SDI	21.66 (12.37 to 32.92)	19.89 (12.06 to 28.25)
Middle SDI	10.18 (5.6 to 16.37)	14.49 (8.43 to 20.84)
Low-middle SDI	3.24 (1.93 to 5)	5.57 (3.3 to 8.59)
Low SDI	1.38 (0.8 to 2.18)	1.87 (1.1 to 2.94)
Diet low in fruits		
Global	8.68 (4.41 to 13.02)	3.73 (1.88 to 5.4)
High SDI	5.41 (2.82 to 7.82)	1.94 (0.98 to 2.85)
High-middle SDI	12.86 (6.47 to 19.85)	3.51 (1.66 to 5.62)
Middle SDI	10.98 (5.35 to 17.08)	4 (1.95 to 5.94)
Low-middle SDI	5.43 (2.87 to 8.11)	5 (2.62 to 7.13)
Low SDI	3.19 (1.67 to 4.82)	2.85 (1.42 to 4.13)
Secondhand smoke		
Global	7.46 (0.95 to 13.72)	3.91 (0.51 to 7.44)
High SDI	8.2 (1.05 to 15.34)	2.61 (0.34 to 5)
High-middle SDI	12.77 (1.59 to 23.83)	7.79 (0.98 to 14.85)
Middle SDI	7.98 (1.06 to 14.8)	4.85 (0.64 to 9.39)
Low-middle SDI	1.97 (0.24 to 3.65)	1.96 (0.25 to 3.74)
Low SDI	0.74 (0.08 to 1.45)	0.73 (0.09 to 1.47)

UI, uncertainty interval; DALYs, disability adjusted life years; SDI, sociodemographic index.

## 4 Discussion

Worldwide, lung cancer ranks as the principal cause of cancer-related mortality, affecting both males and female (10). Although more common in older men, the disease burden among young and middle-aged individuals (15–45 years) is significant (11). Our study reveals multifactorial drivers underlying the observed trends in lung cancer burden among individuals aged 15–45. The global decline primarily reflects comprehensive tobacco control policies implemented following the WHO Framework Convention on Tobacco Control since 2005, with smoking contributing to 23.25 DALYs per 100,000 in 2021, particularly effective in high-SDI countries where smoking-related DALYs decreased from 70.56 to 21.24 per 100,000. Generational smoking pattern shifts explain the faster decline in men (AAPC −1.3% vs. −0.25% in women), while healthcare system improvements have enhanced outcomes in high-SDI regions. However, increasing burden in specific populations reflects environmental pollution patterns, with household air pollution (4.57 DALYs per 100,000) and ambient particulate matter (10.67 DALYs per 100,000) disproportionately affecting low-SDI regions and women. Delayed policy implementation in low-SDI countries and regional industrialization effects, such as East Asia’s high burden (156.8 DALYs per 100,000) from severe air pollution, explain the heterogeneous global patterns. Our findings emphasize the need for targeted strategies, better resource allocation, and specific guidelines for managing lung cancer in younger individuals. These insights are crucial for healthcare practices and future research.

Our focus on the 15–45 age group addresses a critical knowledge gap in lung cancer epidemiology. Unlike older populations where tobacco-related disease patterns are well-established, younger patients represent a heterogeneous group with distinct clinical and epidemiological characteristics that demand separate analysis. The substantial burden we identified—with over 125,000 cases globally in 2021—represents not just individual health impacts but significant societal costs in terms of premature mortality, lost productivity, and family disruption during peak reproductive and career-building years.

### 4.1 Age differences in burden of lung cancer among young and middle-aged people

Our study found a marked decline in the mortality of lung cancer and improved life expectancy in 2021, likely due to advances in medicine, better healthcare accessibility, economic growth, and social protection efforts. The study showed a significant decrease in the prevalence, mortality, and DALYs of lung cancer among individuals aged 15–45, indicating a declining disease burden due to early screening and increased investment in healthcare for young people. From 1990 to 2021, lung cancer prevalence dropped significantly among those aged 35–45, while the decrease was less pronounced among those aged 15–19. This may be due to the 35–45 age group having better access to healthcare and timely treatment. The slower decline in the 30–34 age group may be due to poor health management and unhealthy lifestyles. Factors such as the vaping-associated lung injury (VALI) epidemic, which causes severe illness even in young and healthy individuals, may also contribute to an increased risk of carcinogenesis (12).

### 4.2 Sociodemographic differences in burden of lung cancer among young and middle-aged people

Despite a global decline in lung cancer prevalence among young and middle-aged adults, the trends differ among various sociodemographic index (SDI) groups and across different countries. In 2021, most cases were in middle, high-middle, and high SDI regions, with the highest DALYs in high-middle SDI regions. Our study found that lung cancer prevalence increased with higher SDI, but age-standardized mortality dropped significantly when SDI exceeded 0.7, reflecting socioeconomic progress. High-SDI countries, like the U.S. and Europe, have reduced mortality through comprehensive tobacco control (13, 14). However, in low-middle SDI regions, lung cancer prevalence, mortality, and DALYs have slightly increased over the past three decades, suggesting a need for stronger legal measures, better medical resources, and early screening in these areas.

### 4.3 Sex differences in burden of lung cancer among young and middle-aged people

In people aged 15–45, lung cancer burden is more substantial in men compared to women, with greater age-standardized prevalence, mortality, and DALYs. This is attributed to the fact that men have higher smoking rates. This disparity is understandable, as men tend to smoke more than women, and tobacco serves as a crucial risk determinant in the development of lung cancer. Encouragingly, smoking rates have declined with societal progress; a United States report showed that adult smoking prevalence reached an all-time low of 11.5% in 2021 (15). However, smoking rates have declined, and the reduction in disease burden over the past three decades has been faster in men, despite the overall higher burden. This may be due to higher smoking prevalence in men and greater focus on tobacco control, often neglecting women’s lung cancer risk (9). In 2023, lung cancer among individuals who have never smoked ranks as the fifth most common cause of cancer-related fatalities, particularly in women and Asians (9). Studies show women are more exposed to household pollution on PM2.5 (16), with women in Africa and Asia spending several hours cooking and collecting fuel, increasing their health risks. Our findings show that women aged 15–45 in East Asia have the greatest age-standardized DALYs, highlighting the need for better lung cancer screening and intervention, especially for non-smoking women. Targeted sex-specific treatment strategies are needed for enhanced treatment outcomes.

### 4.4 Risk factors in burden of lung cancer among young and middle-aged people

Smoking ranks as a primary contributor to lung cancer, and it accounts for 30–40% of cancer-related fatalities (17). Our study found that smoking is the primary contributor to DALYs from lung cancer among individuals aged 15–45, highlighting insufficient tobacco control in this population. Many countries, including China (which ratified the WHO Framework Convention in 2005) and the Netherlands (which implemented measures like higher tobacco taxes and smoking bans in 2020–2021), have made strides in tobacco control (18, 19). However, smokeless tobacco use remains a concern, as global efforts to control it lag behind cigarette consumption reduction. Greater commitment to the WHO Framework Convention on Tobacco Control is needed (20). Furthermore, it is crucial for enhancing public health to decrease the exposure of non-smokers to secondhand smoke, which our research has recognized as a notable risk factor for lung cancer.

Household air pollution (HAP), from activities like cooking, heating, and lighting, is a significant lung cancer risk, most notably in low- and middle-income countries (21). The use of solid fuels in poorly ventilated homes contributes to indoor air pollution, emitting harmful particulate matter and gasses similar to tobacco smoke. The World Health Organization makes an estimation that 3.8 million deaths occur each year as a result of HAP (22). In developing countries, lung cancer seems to be a major trigger for premature death among women, who are often primary cooks and non-smokers. This highlights the importance of controlling solid fuel use and addressing lung cancer risks for young and middle-aged women who never smoked and were easily overlooked. HAP is modifiable, and improving fuels, cookstoves, and ventilation can improve health outcomes. Additionally, ambient particulate matter (PM2.5) is a crucial environmental risk element for lung cancer, especially among women and nonsmokers (23). Between 1990 and 2019, lung cancer deaths linked to PM2.5 doubled, especially in middle SDI regions and among men (24). Our findings show that PM pollution has increased the disease burden in low-middle and low SDI regions over the past three decades, despite an overall decline in lung cancer burden.

Our findings support population-specific public health interventions tailored to observed epidemiological patterns. High-SDI countries should continue strengthening tobacco control with focus on emerging products like e-cigarettes and heated tobacco, implement targeted cessation programs for remaining smokers in the 15–45 age group, and address secondhand smoke exposure which contributes 3.91 DALYs per 100,000 globally. Low and low-middle SDI countries require priority interventions addressing clean cooking fuel transitions to combat household air pollution, particularly targeting women who show increasing burden trends, while simultaneously strengthening tobacco control infrastructure including taxation, advertising restrictions, and smoke-free policies, and improving ambient air quality through industrial emission controls and urban planning. The East Asia region specifically needs comprehensive air quality management addressing both ambient and household pollution, enhanced tobacco control targeting high male smoking rates, and gender-specific interventions for the rising burden among never-smoking women. For women in low-SDI regions, interventions should focus on clean cookstove programs and improved household ventilation, education on household air pollution risks during cooking and heating, and healthcare provider training for early recognition of lung cancer symptoms in young women.

Our research encompasses certain limitations. Initially, our dataset originates from the Global Burden of Disease Study 2021, inheriting the typical limitations of the Global Burden of Disease Study, such as inaccuracies in modeling processes that may affect the precision of the data. In addition, based on GBD data, we cannot distinguish between different types of lung cancer and evaluate their epidemiology separately. Moreover, variability in data quality between regions may impact comparability. Finally, more detailed age stratification and consideration of interactions with other stroke risk factors could have improved accuracy.

## 5 Conclusion

From 1990 to 2021, mortality and DALYs from lung cancer among individuals aged 15–45 decreased significantly. While males have had a higher disease burden over the past 30 years, they witnessed a sharper decrease in both prevalence and DALYs compared to females. This trend can be attributed to effective tobacco control measures, improved healthcare accessibility, advances in treatment modalities, and enhanced public health awareness campaigns. However, for women who have never smoked, especially those in countries with high exposure to household pollutants and low SDI, implementing stronger measures to control indoor pollution may be crucial for cutting down the incidence of lung cancer among young and middle-aged women. In our subsequent work, we will be committed to exploring genetic and environmental risk factors in the 15–45 age group.

## Data Availability

The raw data supporting the conclusions of this article will be made available by the authors, without undue reservation.
