# Supplementary material for: Global burden of lung cancer in adolescents and adults aged 15–45: analysis of the global burden of disease study (1990–2021)
Source: Front Med (Lausanne). 2025 Jun 25;12:1600662. doi: 10.3389/fmed.2025.1600662 (PMC12239874; doi:10.3389/fmed.2025.1600662)

Supplementary Figure 1.The changes in the proportion of prevalence cases(A), mortality(B) and disability-adjusted life years among lung cancer patients aged 15–45 years to the overall lung cancer patients from 1990 to 2021.

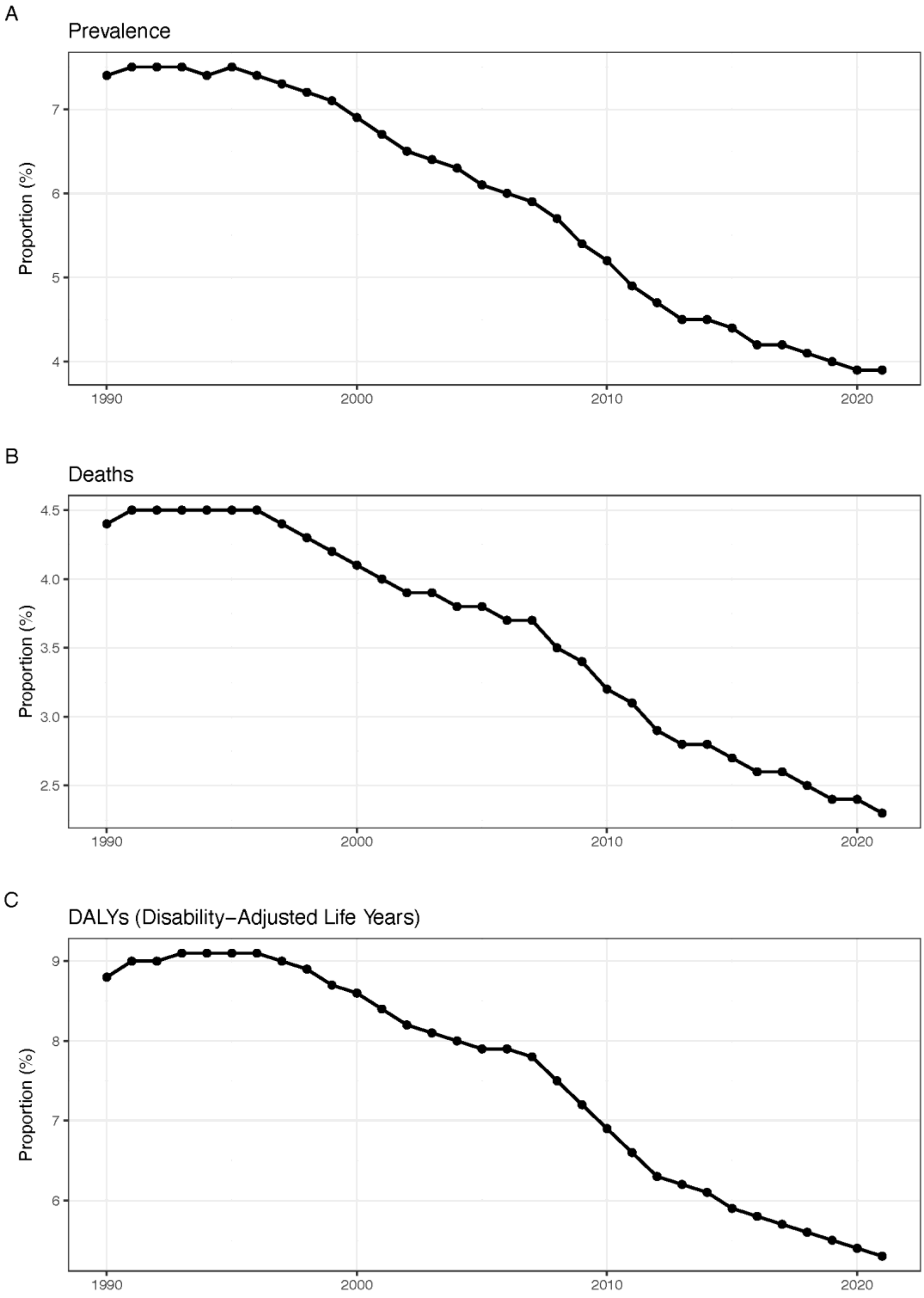

Supplementary Figure 2.Temporal trend of age-standardized prevalence, mortality and disability-adjusted life years for lung cancer patients aged 15–45 years and overall lung cancer patients from 1990 to 2021.

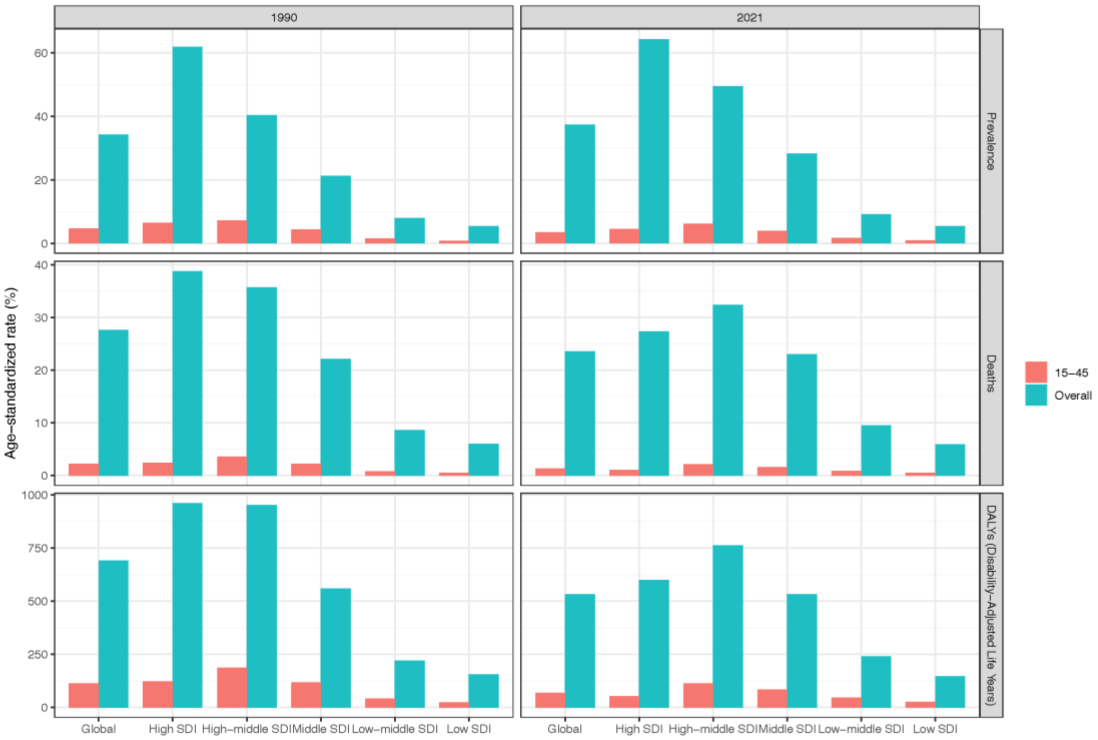

Supplementary Figure 3. Average annual percent changes of age-standardized mortality and disability-adjusted life years of lung cancer in young and middle-aged people from 1990 to 2021 at socio-demographic index levels by sex.

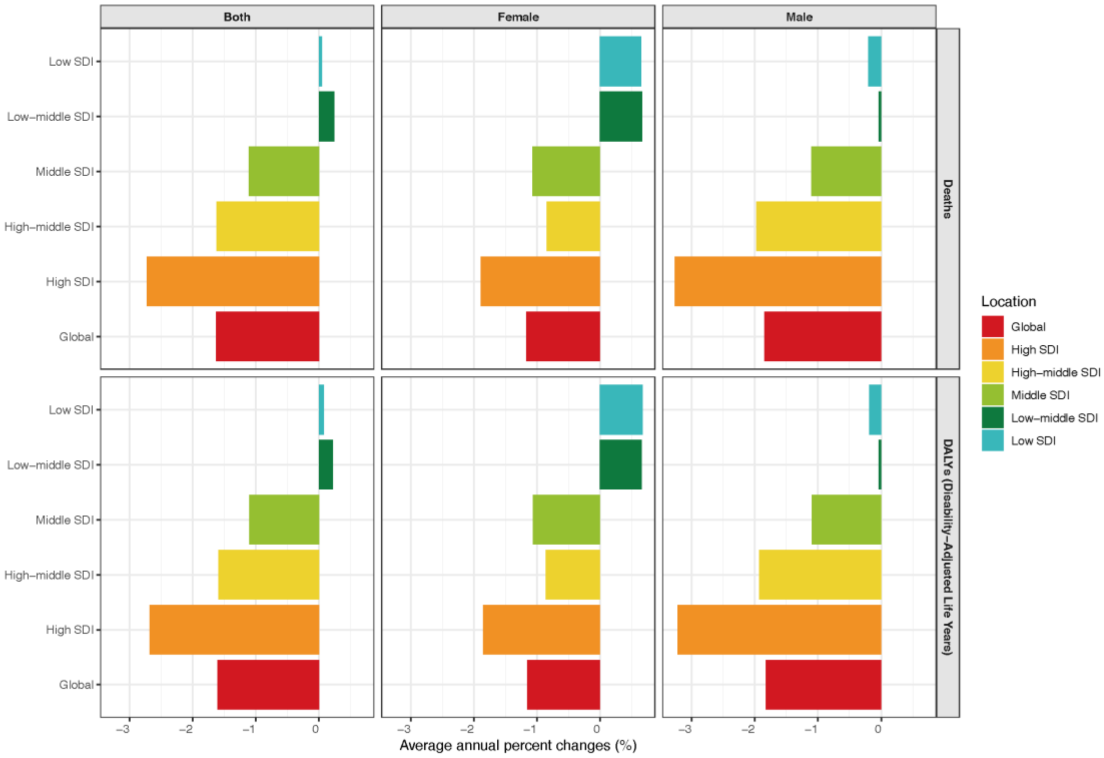

Supplementary Figure 4. Average annual percent changes of age-standardized prevalence, mortality and disability-adjusted life years of lung cancer in young and middle aged people from 1990 to 2021 by sex and age.

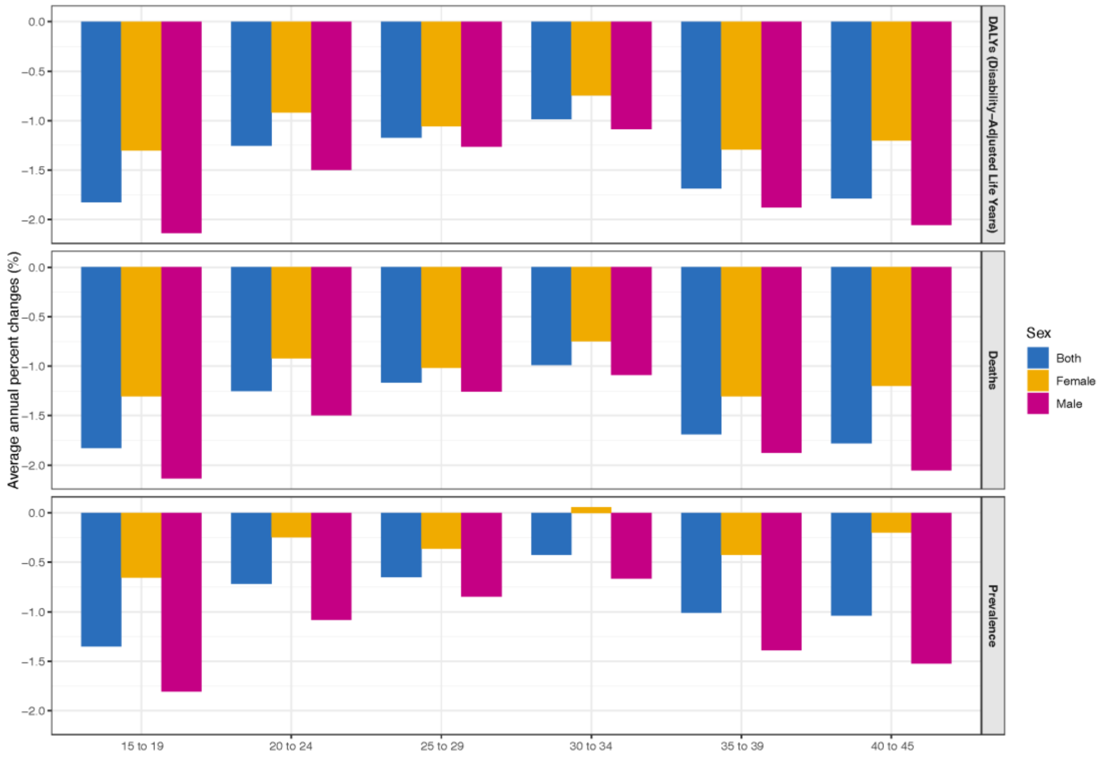

Supplementary Figure 5.Temporal trend of age-standardized prevalence, mortality and disability-adjusted life years of lung cancer aged 15–45 years and overall lung cancer patients from 1990 to 2021 at global and sociodemographic index levels.

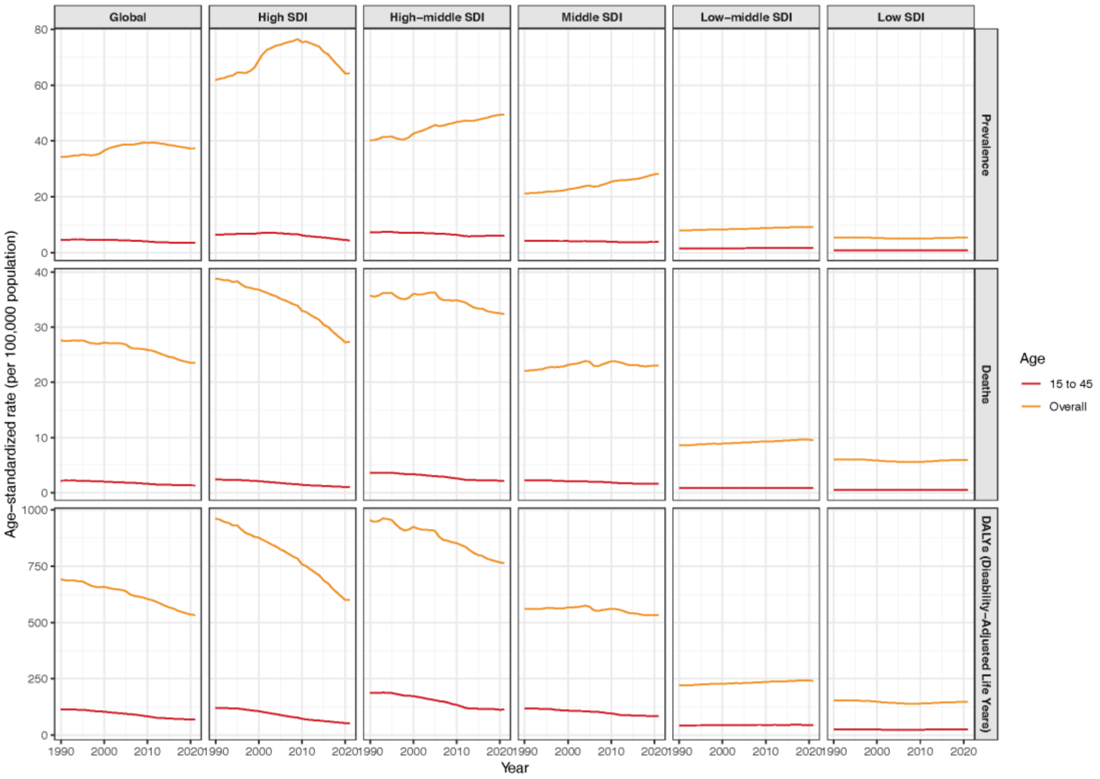

Supplementary Figure 6. Average annual percent changes of age-standardized prevalence, mortality and disability-adjusted life years of lung cancer in people aged 15–45 years from 1990 to 2021 at regions levels.

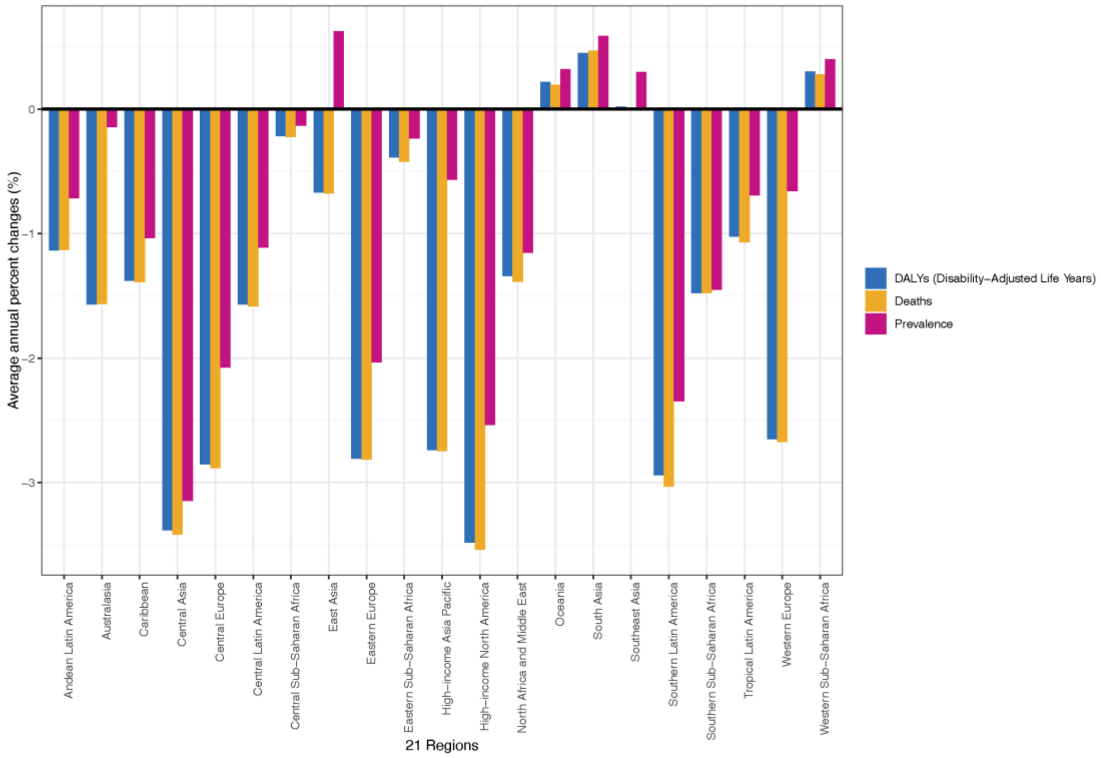

Supplementary Figure 7. Average annual percent changes of age-standardized mortality and disability-adjusted life years of lung cancer in young and middle aged-people aged 15–45 years from 1990 to 2021 at regions levels by sex.

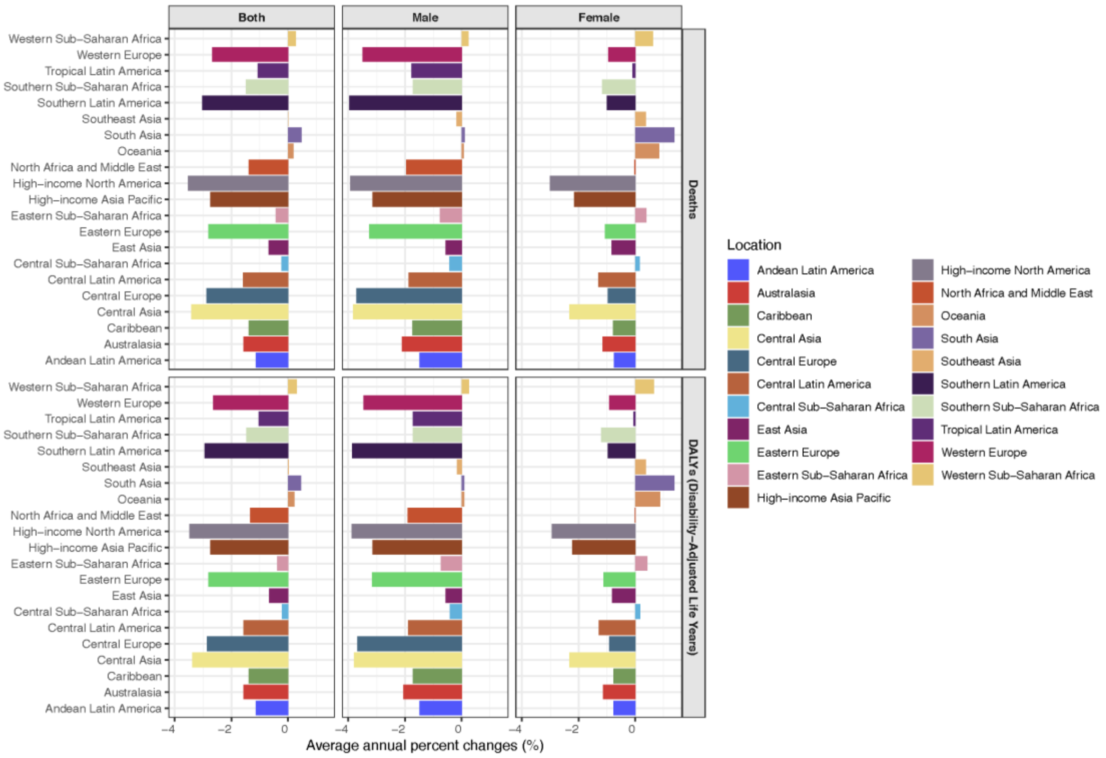

Supplementary Figure 8. Map showing average annual percentage change in global mortality among people with lung cancer aged 15–45 years, 1990–2021.

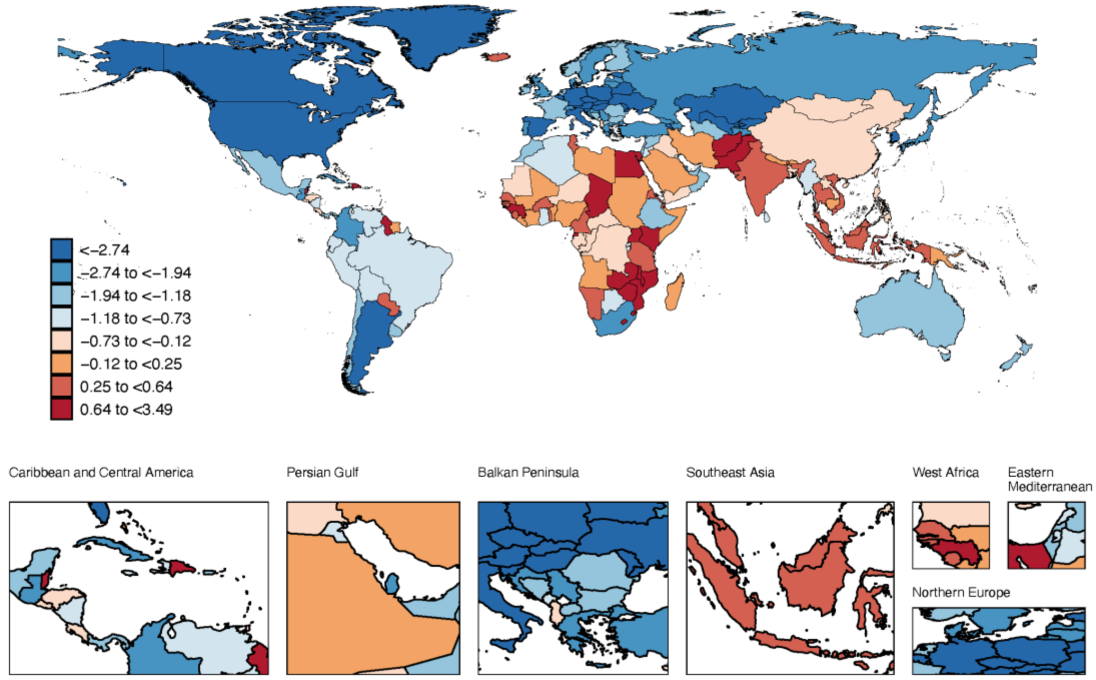

Supplementary Figure 9. Map showing average annual percentage change in global DALYs among people with lung cancer aged 15–45 years, 1990–2021.

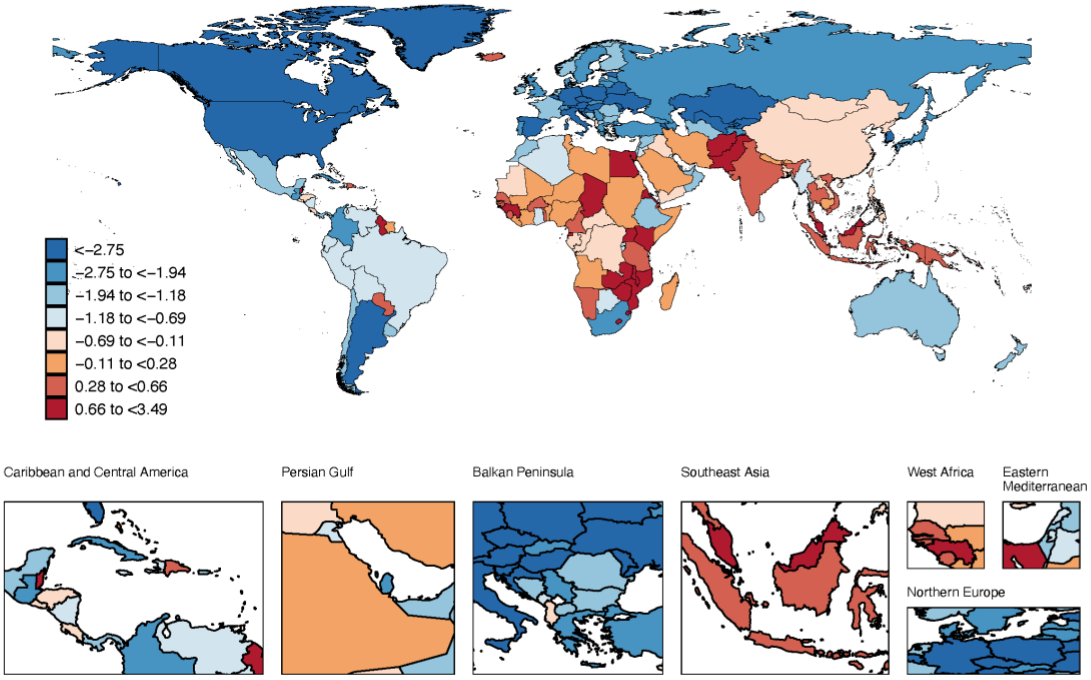

Supplement: Supplementary file 1 [file Data_Sheet_1.PDF]
